# Identification of four immune subtypes in locally advanced rectal cancer treated with neoadjuvant chemotherapy for predicting the efficacy of subsequent immune checkpoint blockade

**DOI:** 10.3389/fimmu.2022.955187

**Published:** 2022-09-27

**Authors:** Le He, Min Jin, Dan Jian, Bo Yang, Nan Dai, Yan Feng, He Xiao, Dong Wang

**Affiliations:** ^1^ Cancer Center, Daping Hospital and Army Medical Center of People's Liberation Army (PLA), Third Military Medical University (Army Medical University), Chongqing, China; ^2^ Department of Gastroenterology, Chongqing General Hospital, Chongqing, China

**Keywords:** rectal cancer (RC), immune checkpoint inhibitors, immunophenotyping, genomics, neoadjuvant (chemo)radiotherapy

## Abstract

**Introduction:**

Neoadjuvant chemoradiotherapy (nCRT) is the foundation treatment for locally advanced rectal cancer (LARC). The nCRT can improve the efficacy of immunotherapy because of its in situ vaccine effect.

**Objective:**

The aim is to identify stable and reliable transcriptome signatures to predict the efficacy of immune checkpoint blockade (ICB) in patients with LARC.

**Methods:**

Immunophenotyping was established using xCell immune cell infiltration abundance and consistent clustering in GSE39582 and verified in several data sets. The effects of immunophenotyping, follicular regulatory T cells, tumor-associated fibroblasts (CAFs), and tertiary lymphoid structure (TLS) signatures on the efficacy of ICB were analyzed using IMvigor210, GSE91061, and an independent Daping Hospital (DPH) cohort.

**Results:**

There are four stable and repeatable immune subtypes in rectal cancer, among which C1 is a low immune infiltration type, C2 is a high interstitial infiltration type, C3 is a high immune infiltration type, and C4 is an ion channel type. C2 is mainly characterized by high infiltration of CAF. C3 is characterized by high infiltration of cytotoxic T lymphocytes, high expression of PD-L1 and TLS. In rectal cancer patients receiving nCRT, immunophenotyping was not significantly associated with pathological remission rate, but immunophenotyping was an independent prognostic factor of RFS. In IMvigor210 patients treated with atezolizumab, the pathological remission rates of C1, C2, C3, and C4 were 23.86%, 10.94%, 33.33%, and 23.08% respectively (χ2 = 8.981, P = 0.029), which were 11.76%, 50.00%, 42.86%, and 0.0% respectively in the GSE91061 patient treatment with nivolumab (Fisher’s exact probability, P = 0.018). Both follicular regulatory T cells and CAF showed a further impact on the ICB therapeutic efficacy of C2 and C3 subtypes. Additionally, both the GSE91404 and DPH cohorts showed that nCRT treatment induced a significant increase in the expression of TNFRSF9 and the abundance of macrophages in the C3 subtype.

**Conclusion:**

Our data suggest that there are four immune types of rectal cancer, which are related to the prognosis of patients. Among them, C3 and some C2 subtypes represent the patients who may benefit from ICB after nCRT treatment.

## Introduction

Neoadjuvant chemoradiation therapy (nCRT) and total mesorectal excision are commonly incorporated into the multimodal treatment of locally advanced rectal cancer (LARC). nCRT has been shown to reduce tumor burden, improve operative procedures and prevent local tumor recurrence (1–3). nCRT can induce an in situ vaccine effect and promote antitumor immunity. Therefore, a large number of preclinical and clinical studies have explored the feasibility of nCRT combined with immune checkpoint blockade (ICB) in LARC (4–7). There is evidence suggesting that the combination of CTLA-4 and PD-1 with chemoradiotherapy is the most effective combination therapy strategy (6). However, because of the heterogeneity of the tumor, it is speculated that this combination therapy is not effective for all patients in clinical studies. Therefore, there is an urgent clinical need to screen patients who most likely benefit from the treatment of nCRT combined with ICB based on biomarkers, especially by transcriptomic signatures, which remains a huge challenge. The main reasons are as follows: 1) there is still no universally applicable biomarkers for predicting benefit of ICB treatment across all tumor types. Although PD-L1 expression, tumor mutation burden (TMB), IFNγ-related T-cell-inflated gene expression profile (8) and tumor immune dysfunction and exclusion (TIDE) (9) have been established, these biomarkers and transcriptomic signatures have only low predictive power in independent validation (10). Second, the regulation of nCRT in the immune microenvironment is complex. In addition to the infiltration of CD8+ T lymphocytes induced by nCRT, it can also actively promote the infiltration of a large number of myeloid inhibitory cells and regulatory T cells (6). Recent studies have shown that other cell populations or tissue structures in the tumor microenvironment, such as tumor associated fibroblasts (CAF), follicular regulatory T cells, tertiary lymphoid structures (TLS) or B cells, have an important impact on the function of CD8+ T cells (11–13). These results suggest that different cell populations of the tumor microenvironment participate in the regulation of tumor immune microenvironment (TIME) heterogeneity and lead to different ICB responses. Therefore, a single immune marker cannot fully satisfy the response prediction or prognosis of patients treated with ICB under the background of different immune tumor microenvironments.

Molecular subtyping can identify tumor subsets with the same biological characteristics, which has become one of the main methods to overcome the tumor heterogeneity. In colorectal cancer, consensus molecular subtypes (CMSs) and colorectal cancer typing (CRCAssigner) based on mRNA expression profiles predict the efficacy of bevacizumab and anti-EGFR in RAS wild-type advanced colorectal cancer (14, 15). Similarly, immune subtypes (ISs) depending on immune cell abundance have revealed good capacity to discriminate patients who response to ICB from others in melanoma, sarcoma, and glioma (16–19). Therefore, we jointly used the immunophenotyping of colon cancer and transcriptomic signatures of follicular regulatory T cells, tumor associated fibroblasts (CAFs) and tertiary lymphoid structures (TLSs) to evaluate ICB response and prognosis, so as to screen the LARC patients who most likely benefit from nCRT combined with ICB.

## Methods and materials

### Datasets retrieving and preprocessing

Raw data of transcriptional expression (CEL files) profiled with Affymetrix were downloaded from GEO (Gene Expression Omnibus). Function “rma” or “threestep” in the limma package was used to obtain the expression matrix at the probe level with default parameters. For raw data of transcriptional expression profiles generated with Agilent, functions “backgroundCorrect” and “normalizeBetweenArrays” were used to correct the background and normalize the data. Function “neqc” was used for preprocessing data profiles with Illumina HumanHT-12 microarrays. Features expressed in at least 30% of samples in each cohort with “Detection Pval <0.05” were retained for further analysis. For genes represented by several probes, the probe with the maximum interquartile range was finally selected as the expression of that gene. RSEM values on the log2 scale of the transcriptional profile of TCGA COADREAD (version 2017-09-08) and phenotype file (version 2019-12-06) were obtained from https://xenabrowser.net/datapages. Raw count of RNAseq and responsiveness along with other clinical characteristics of metastatic urothelial cancer treated with atezolizumab monotherapy were extracted from the R package “IMvigor210CoreBiologies.” The genes were retained that had at least 10 counts for 50% of the samples and converted into FPKM by using the annotation file “Biomart.annotations.hg38” and the function “countToFPKM” from the R package “fpkm” (20). FPKM values of GSE91061 were downloaded from GEO deposition and used for statistical analysis without further modification.

The Daping Hospital cohort (DPH) was comprised of resected tissues from nine LARC patients with complete pathological response (pCR), nine without pathological response (npCR), and biopsy tissues from nine LARC patients prior to nCRT. RNAseq of DPH cohort tissues was performed with Illumina Hiseq 2500. FPKM was obtained from raw count after being adjusted for length of exons and library size. Information about datasets used in this study was summarized in **Supplementary Table 1**.

The studies involving human participants were reviewed and approved by ethics committee of Daping Hospital. The Helsinki Declaration was followed strictly in this study.

### Development of immune subtypes and prediction

The immune cell composition of 566 colon tumor tissues deposited in the GSE39582 dataset was evaluated with “xCellAnalysis,” which provides abundance scores for 64 immune cell populations (21). Consensus clustering of 64 immune cells across 566 samples was performed through the function “ExecuteCC” in the R package “CancerSubtypes” setting parameter “clusterAlg=‘pam’, distance=‘pearson’” after z-value transformation of abundance scores in each sample. An average z-value in each of the 64 immune cell populations in each subtype was calculated as the centroid of each immune subtype to predict the immune subtypes of other cohorts and platforms. To predict de novo immune subtypes of additional cohorts, z-values of abundance scores of 64 immune cell populations were computed. Each sample was assigned to the closest immune subtype based on Pearson coefficients with the centroid of each immune subtype. However, samples were assigned unclassified if the maximum Pearson correlation coefficient was less than 0.15 or the difference between the maximum and the second maximum Pearson correlation coefficient was less than 0.06. These criteria were similar to those used in the CRCA classification system (22). The centroids of 64 immune cell populations derived from GSE39582 are shown in **Supplementary Table 2**.

### GSVA analysis to infer characteristics of each immune subtype

Gene set variation analysis was used to explore the immune characteristics of each immune subtype in GES39582. The c5 gene set collection of MSigDB was used to calculate the enrichment score for a total of 10,485 pathways after infiltrating pathways with a number of component genes of less than 10 or greater than 500 genes (23, 24). The parameter mx.diff was set to TRUE in all GSVA analysis. Stepwise multiple comparisons of enrichment scores returned from GSVA among immune subtypes were performed with the R package “limma”. The pathways with a P-value adjusted for multiple comparisons of less than 0.05 in all comparisons were identified. The top 10 pathways with the maximum enrichment score in each immune subtype were selected to represent the immune features of that immune subtype and are illustrated in the heatmap.

### Estimation for the activity of follicular regulatory T cells in tumor tissues

Recently, Eschweiler identified an important regulatory subset of FOXP3-espressing CD4+ T cells that are highly suppressive to affect T cells and co-express BCL6 and/or CXCR5, indicated as follicular lineage (12). To establish the relevant abundance of such a subset of follicular regulatory T cells (Tfr cells) to Treg cells (TFRscore), the differentially expressed genes (DEGs) between Tfrs (CD4+CXCR5+GITR+, n = 10) and Tregs (CD4+CD25+CXCR5−, n = 10) were first identified through the R package “edgeR” using raw count in GSE132295. The centroid of these DEGs in Tfr and Treg cells was calculated with a transformed z-value of the FPKM value. TFRscore of a given tumor tissue sample was calculated based on formula (6) proposed by Budcizies by using centroids established above and z-value of gene expression of that tumor tissue (25). The gene list of DEGs identified in GSE132295 and the centroids of Tfr and Treg cells are shown in **Supplementary Table 3**.

### Scores for tertiary lymphoid structures and TGFβ-induced genes

The score for tertiary lymphoid structure (TLSscore) was calculated based on the average of the seven marker genes proposed by Cabrita in the original article. The marker genes for TLSscore included CCL19, CCL21, CXCL13, CCR7, CXCR5, SELL, and LAMP3 (13). A pan-fibroblast TGFβ response signature consisting of 19 genes commonly induced by TGFβ in isolated fibroblasts was used to calculate the fibroblast TGFβ response score (FTBRS). FTBRS was calculated as the average expression of the pan-fibroblast TGFβ response signature (11).

### Statistical analysis

The abundance of 19 immune cell populations was evaluated with ConsensusTME (26). A Kruskal–Wallis test with multiple comparisons was used to evaluate differences in TFRscore, TLSscore, FTBRS, and abundance of infiltrated immune cells among immune subtypes. The relationship between pathological response and MSI status, MMR status, and immune subtypes was estimated with the chi-square test or Fisher’s exact probability. Univariate and multivariate Cox regression were used to reveal independent prognostic factors for recurrent-free survival, progression-free survival, and overall survival. Kaplan–Meier curves along with a log-rank test were used to evaluate differences in OS among immune subtypes. All the tests were two-sided. A P-value of less than 0.05 was considered statistically significant.

## Results

### Four immune subtypes widely exist in colorectal cancer, which are related to RFS

**Figure 1:**
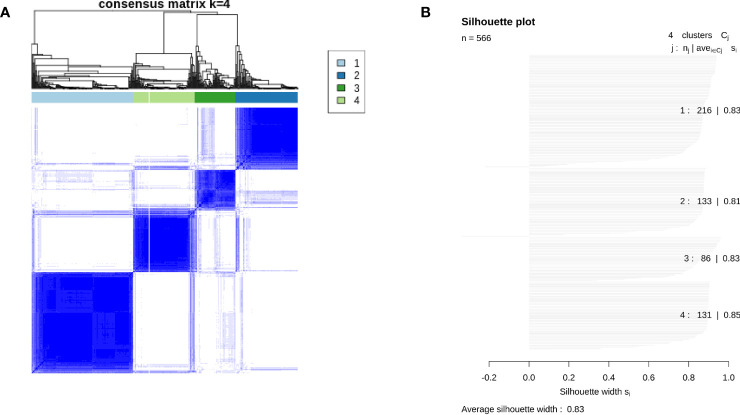
Results of consensus clustering across 566 colon tumor tissues in GSE39582. **(A)** Consensus matrix showing clustering consensus of each case in individuals of four immune subtypes. **(B)** Silhouette plot showing average silhouette score of each immune subtype and the whole population in GSE39582.

**Figure 2:**
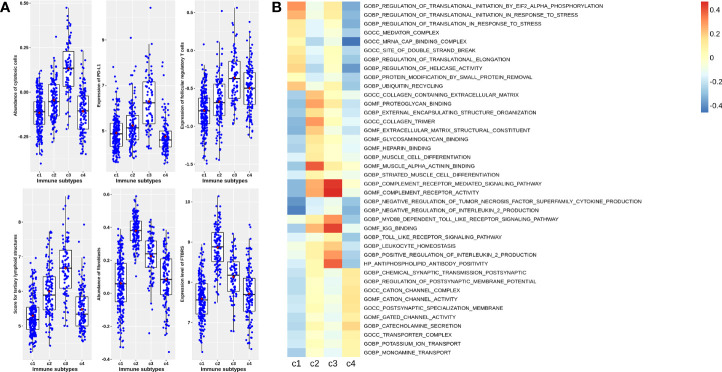
Characteristics of four immune subtypes in GSE39582. **(A)** Scatter plots overlapped with box plots illustrating differences in six major immune related scores among four immune subtypes. The red diamonds in each box indicate mean value. **(B) **Results from GSVA analysis showing ten major significantly enriched pathways in each immune subtype.

Unsupervised cluster analysis found four highly consistent immune subtypes could be obtained in GSE39582 (**Figures 1A, B**). The accuracy was evaluated as 96.44% through the Pearson correlation coefficient method within GSE39582 (see Methods and materials). Analyzing the characteristics of the four immune subtypes, it was found that the proportions of mismatch repair defect (dMMR) in C1, C2, C3, and C4 subtypes were 0.075, 0.0569, 0.3836, and 0.2033, respectively, with significant differences (χ2 = 52.629, df = 3, P= 2.2 × 10−11). Among them, C3 is the immune subtype with the highest cytotoxic T lymphocytes, PD-L1 expression, TLS score, and TFR score value (**Figure 2A**). C2 also contained some expression of cytotoxic T cells and PD-L1, but fibroblast abundance and FTBRS were the highest in C2. The cytotoxic T lymphocytes, PD-L1 expression, TLS score, and TFR score value were low in C1. GSVA analysis showed that the C1 subtype is primarily based on the translation initiation regulatory pathway, C2 is the extracellular matrix or collagen-related pathway, C3 is mainly marked by the activation of IL2, which is the main marker of CD4+T and CD8+T activation, and C4 is mainly based on the activation of ion channel activity (**Figure 2B**). The above results showed that C1 was a low-immune infiltration type, C2 was a high-interstitial infiltration type, C3 was a high-immune infiltration type, and C4 was an ion channel type. The four immunophenotypes can be verified in GSE14333, GSE 26682, GSE 87211, and TCGA COADREAD colorectal cancer sample data (**Supplementary Figure 1**).

**Figure 3:**
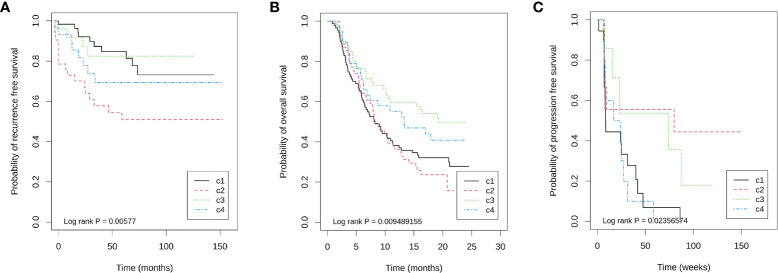
Kaplan–Meier plots showing differences in prognosis within four immune subtypes in three cohorts. **(A)** GSE87211 for recurrence free survival, **(B)** IMvigor210 for overall survival, and **(C)** GSE19106 for progression free survival.

The above results are verified by GSE87211. The Chi-square test showed that there was no significant difference in the pathological remission rate among the four immune types (χ2 = 3.857, df = 3, P = 0.277), and the pathological remission rates of C1, C2, C3, and C4 subtypes were 0.55, 0.418, 0.633, and 0.467, respectively. Survival analysis revealed that immunophenotyping was an influencing factor of RFS after nCRT for rectal cancer, and the risk of recurrence of C2 relative to C1 was significantly increased by 3.2 times (c2 vs c1, HR = 3.239, 95% CI: 1.494–7.022, P = 0.003), but C3 and C4 had no significant effect relative to C1 (HR = 0.921, 95% CI: 0.289–2.937, P = 0.889; HR = 1.660, 95% CI: 0.655–4.208, P = 0.285) (**Figure 3A**). After adjusting for age, sex (male vs female), KRAS mutation (mutation vs wild), neoadjuvant therapy regimen (5-FU + oxaliplatin + RT ± cetuximab vs 5-FU + RT), residual lymph nodes after neoadjuvant therapy (positive vs negative), and pathological remission (remission vs non-remission), C2 was still a risk factor for recurrence (C2 vs C1, HR = 2.752, 95% CI: 1.241–6.105, P = 0.013) (**Supplementary Table 4**). These results suggest that rectal cancer has similar immunophenotyping characteristics as colon cancer, in which C3 is an immune infiltrative type, C2 is a mesenchymal cell infiltrative type, and C1 is an immune desert type. In rectal cancer patients receiving nCRT, there is no correlation between immunophenotyping and pathological remission rate, but it can be used as an independent prognostic factor of RFS.

### Effects of immune subtypes, TFR follicular regulatory cells, and FTBRS fibroblasts on ICB therapy

We found four immune subtypes in rectal cancer, but the relationship between these immune subtypes and the efficacy of ICB therapy is unclear. Because of the lack of data on ICB therapy in rectal cancer, we attempted analyzing the relationship by analyzing the effects of immunophenotyping on the efficacy of immunotherapy for bladder cancer and melanoma.

IMvigor210 is a database for advanced bladder cancer treated with atezolizumab. A total of 298 samples were available for analysis. Among them, there were 88, 64, 60, 39, and 47 cases of C1, C2, C3, C4, and unclassified (UC), respectively, with an unclassified proportion of 15.77%. Among 251 known immunophenotyping samples, the immune grades (IC0, IC1, and IC2) obtained by PD-L1 immunohistochemistry were significantly different among the four immunophenotyping (χ2 = 56.363, df = 6, P = 2.46 × 10−10), in which the proportions of C3 in IC0, IC1 and IC2 were 4.29% (3/70), 15.05% (14/93), and 48.86% (43/88), respectively. Importantly, the objective remission rate (ORR) of ICB therapy was significantly different among different immune subtypes. The ORR of C1, C2, C3, and C4 were 23.86% (21/88), 10.94% (7/64), 33.33% (20/60), and 23.08% (9/39), respectively (χ2 = 8.981, df = 3, P = 0.029). Additionally, immunophenotyping is also a prognostic factor of OS, in which C3 has the longest overall survival time, C4 is the second, and C1 and C2 are the worst (**Figure 3B**).

However, there was no significant difference in TFRscore and FTBRS between the non-remission group and the good remission group (median: −1.014 vs −1.072, Kruskal–Wallis χ2 = 0.029, df = 1, P = 0.864; Median: 2.157 vs 2.001, Kruskal–Wallis χ2 = 2.765, df = 1, P = 0.096), suggesting that fibroblasts seem to have little effect on the efficacy of ICB therapy in advanced bladder cancer. Using the 66.66% quantile value of TFRsocre as the cut-off value, it was found that TFRscore was the prognostic factor of OS only in the c3 subtype, and OS was significantly longer in patients with high expression than in patients with low expression. These results suggest that the role of follicular regulatory T lymphocytes is different in different immune-infiltrating subtypes.

GSE19106 is a database of advanced melanoma treated with nivolumab, including 109 biopsy samples before and after treatment. The classifications of C1, C2, C3, C4, and UC were 34, 15, 23, 19, and 19 cases, respectively, and the unclassified proportion was 16.51%. Among 42 pre-treatment samples that could be classified, the remission rates of four immune subtypes C1, C2, C3, and C4 were 11.76% (2/17), 50.00% (4/8), 42.86% (3/7), and 0.0% (0/10), respectively (Fisher’s exact probability, P = 0.018). Similarly, the PFS of C2 and C3 is longer (**Figure 3C**). Because the sample size of each immune subtype is small, Cox regression of OS cannot be performed in each subtype. However, to show the impact of TFR score and FTBRS on the efficacy of nivolumab in different subtypes, only the expression differences in these two indices between remission and non-remission groups in the C2 and C3 groups were analyzed. The results show that only the TFR score was significantly different between the remission and non-remission groups in C2 subtypes. The TFR score in the remission group was significantly higher than that in non-remission cases. However, in both C2 and C3 subtypes, FBRS in the non-remission group was higher than that in the remission group (**Supplementary Figure 3**).

The above analysis of bladder cancer and melanoma shows that C3 and C2 subtypes can benefit from ICB treatment. However, follicular regulatory T cells and fibroblasts have further regulatory effects on ICB efficacy on the basis of immunophenotyping.

### nCRT treatment can activate the antitumor immunity of rectal cancer

GSE94104 is a database of 40 paired samples before and after neoadjuvant treatment with 45 Gy + 5-FU/carboplatin. A total of 20 cases in the paired samples can be classified. The composition of the four immune types before and after nCRT treatment is shown in **Table 1**. There were nine cases of C1 before treatment, three cases of C1 transformed into C3, and two cases of C1 transformed into C2 after treatment. After treatment, two cases of C4 changed to C2 and one case changed to C3, indicating that nCRT can increase the tumor-infiltrating lymphocytes. Additionally, the proportion of C2 after radiotherapy and chemotherapy was as high as 45% (9/20), indicating that radiotherapy and chemotherapy can also induce an increase in mesenchymal cells.

**Table 1:** Immune subtypes in matched samples before and after nCRT in GSE94104.

Pre nCRT/Post nCRT	c1	c2	c3	c4	Total
c1	3	2	3	1	9
c2	1	2	0	0	3
c3	0	3	0	0	3
c4	0	2	1	2	5
Total	4	9	4	3	20

**Figure 4:**
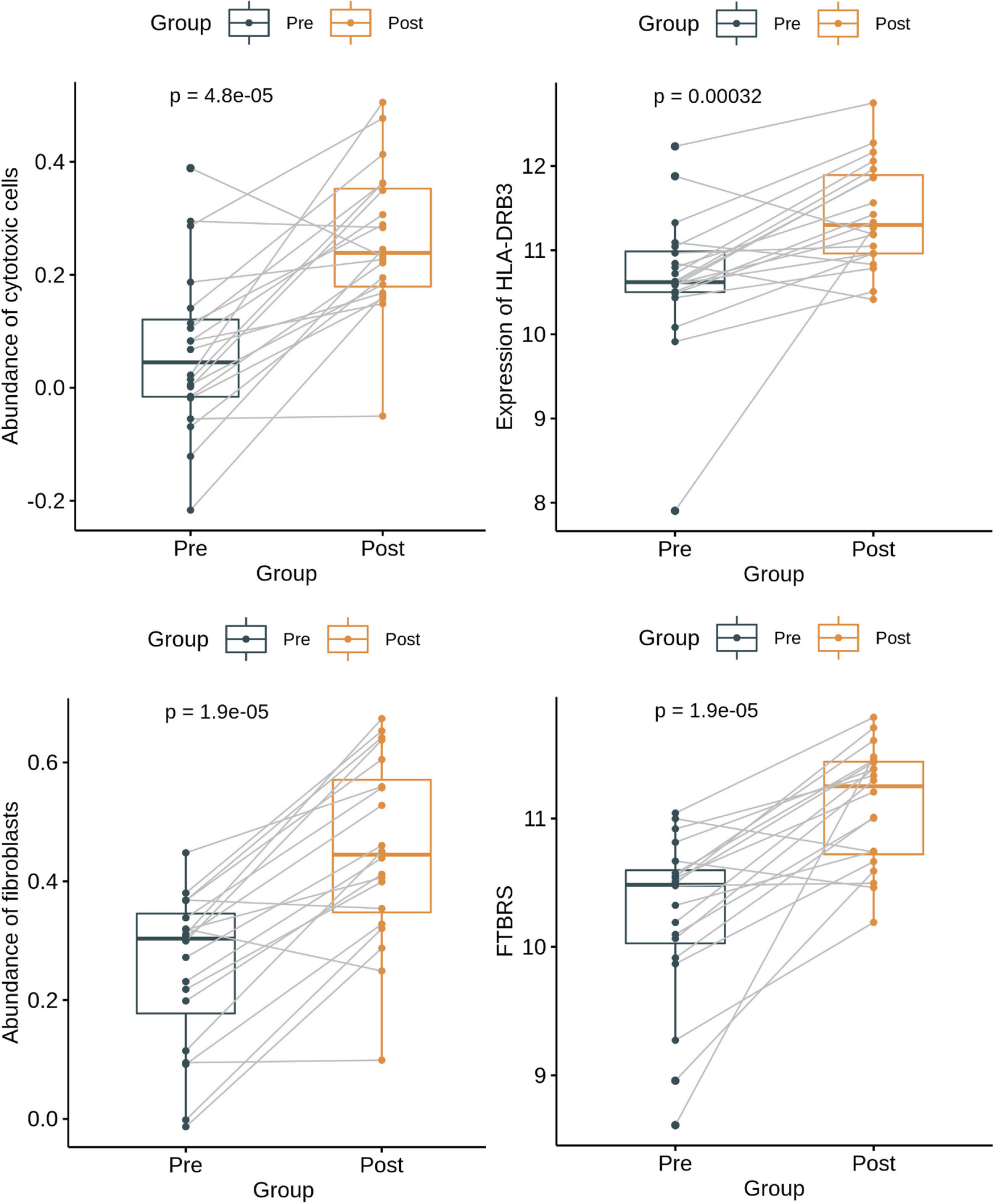
Paired box plots showing changes in abundance of cytotoxic cells, expression level of HLA-DRB3, abundance of fibroblast cells, and FTBRS between after nCRT and before nCRT.

**Figure 5:**
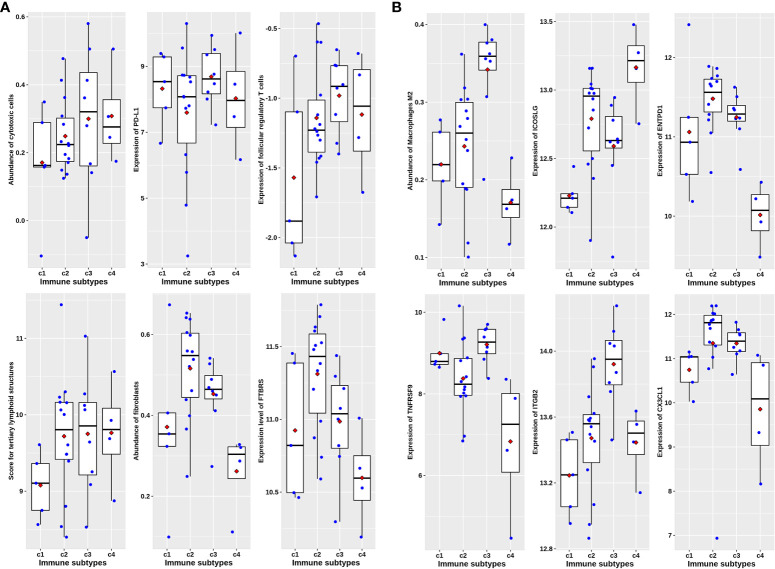
Immune characteristics among four immune subtypes in post-nCRT samples in GSE94104. **(A)** Scatter plots overlapped with box plots illustrating differences in six major immune related scores among four immune subtypes without statistical significance. **(B)** Scatter plots overlapped with box plots illustrating significant differences in six immune related scores among four immune subtypes. The red diamonds in each box indicate mean value.

The analysis of paired samples of 20 cases before and after neoadjuvant treatment showed that the expression of cytotoxic T cells and class II antigen-presenting molecules after nCRT treatment was significantly higher than that before treatment (**Figure 4**). Among the four immune subtypes after nCRT, there were no significant differences in the expression of cytotoxic T lymphocytes or PD-L1 (**Figure 5A**). Only fibroblasts and FTBRS scores were the highest and significantly different in C2. However, we found that the C3 expression of TNFRSF9 and ITGB2 in M2 macrophages was significantly higher than that in other subtypes (**Figure 5B**), in which TNFRSF9 was a marker of regulatory T-cell receptor activation. These results suggest that nCRT promotes antitumor immunity, particularly activated antigen presentation and the infiltration of effector T lymphocytes. However, due to the influence of radiotherapy and chemotherapy, the immunophenotyping characteristics after nCRT treatment are different from those before treatment.

**Figure 6:**
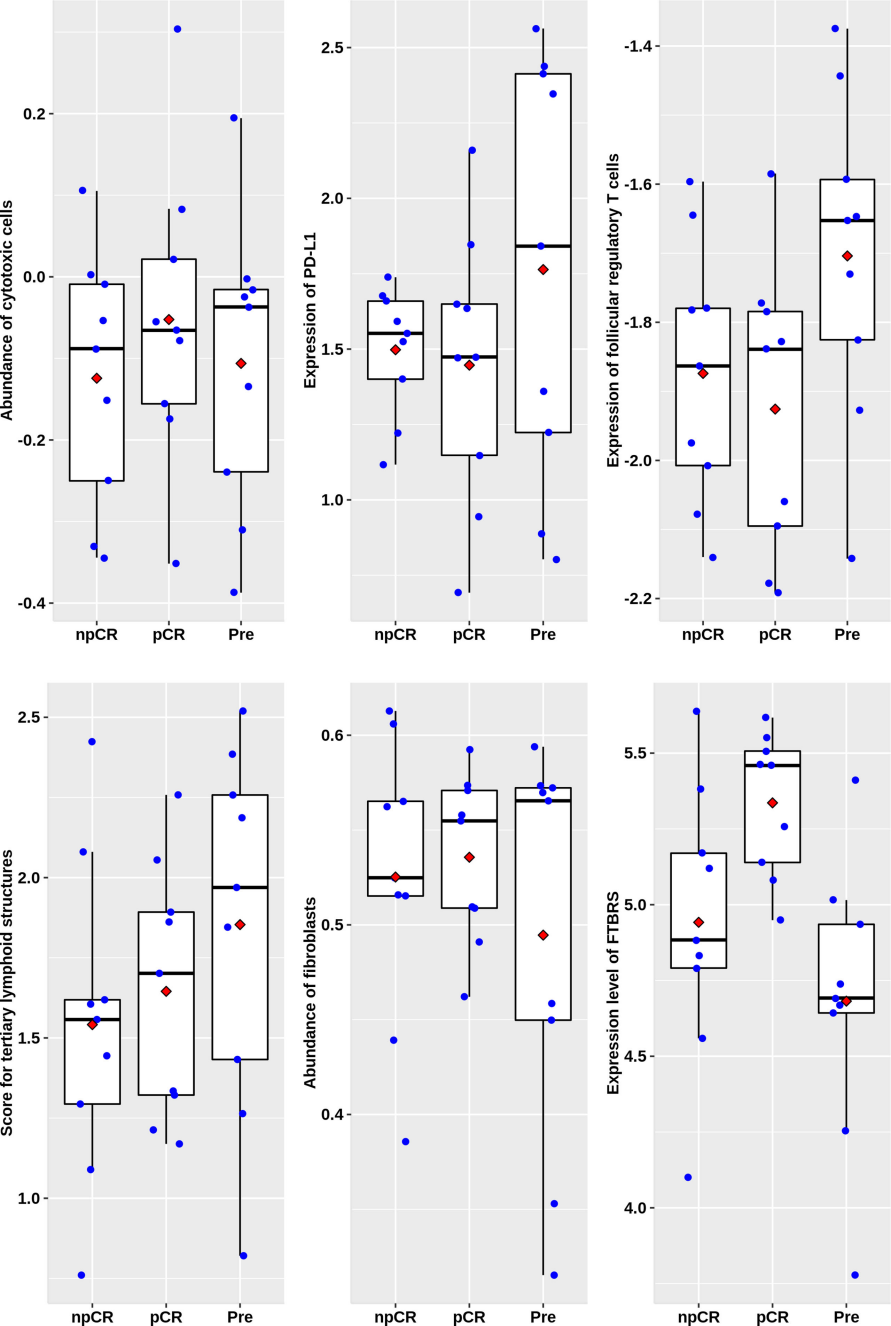
Scatter plots overlapped with box plots illustrating differences in six major immune related scores among four immune subtypes in DPH cohort. npCR indicates samples with non-pathological complete response after nCRT treatment, pCR indicates samples with pathological complete response after nCRT treatment and Pre stands for samples biopsied before nCRT.

**Figure 7:**
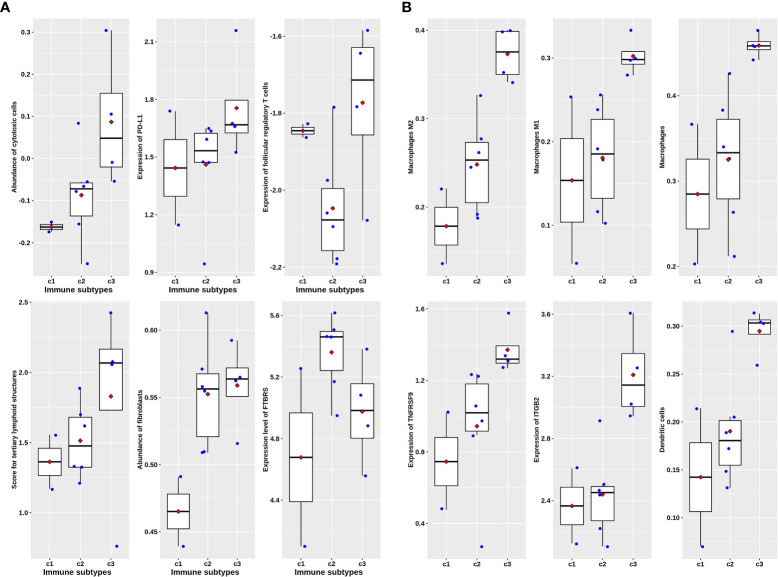
Scatter plots overlapped with box plots illustrating immune characteristics among three immune subtypes available after nCRT treatment in DPH cohort. **(A)** Six major immune related scores without statistical significance. **(B)** Six immune related scores with statistical significance revealed by Kruskal–Wallis H test.

In the 27 rectal cancer samples we collected and sequenced (DPH data set), there were no significant differences in cytotoxic T lymphocytes, PD-L1 expression, follicular regulatory T cells, and TLS indicators among non-chemoradiotherapy (uCRT), non-pathology complete remission (nPCR), and PCR groups (**Figure 6**), and only the FTBRS value was significantly higher in the PCR group than that in uCRT samples (adjusted P = 0.002). Among the 27 samples, only 18 had immunophenotyping. In the six cases without nCRT, there were one, two, two, and one cases of C1, C2, C3, and C4 subtypes, respectively, while in the 12 cases after nCRT, there were two, six, four, and zero cases, respectively (Fisher’s exact probability, P = 0.774). Among the samples that could be classified after treatment, the PCR rates of C1, C2, and C3 subtypes were 50.0% (1/2), 83.3% (5/6), and 25.0% (1/4), respectively, which did not reach statistical significance (Fisher’s exact probability, P = 0.192). Although there was no significant correlation with pathological remission before and after treatment, the expression of cytotoxic T cells, PD-L1 expression, TLS, and FTBRS increased in C1, C2, and C3 subtypes after treatment, which was consistent with the trend in the analysis of GSE39582 and GSE87211 (**Figure 7A**). Statistically significant were mainly macrophage and dendritic cell abundance, TNFRSF9 and ITGB2 expression, which were consistent with the results of GSE94104 (**Figure 7B**).

## Discussion

Rectal cancer is a heterogeneous disease at the genetic and molecular levels; both aspects have major repercussions on the tumor immune context. While microsatellite status and tumor mutational load have been associated with the response to immunotherapy, the presence of TILs is one of the most powerful prognostic and predictive biomarkers. Yet, the majority of rectal cancers are characterized by microsatellite stability, low-tumor mutational burden, and poor T-cell infiltration. These patients can also benefit from ICB treatment, suggesting that biomarkers cannot fully reflect the immune microenvironment characteristics of rectal cancer (27). Additionally, radiotherapy and chemotherapy can lead to the remodeling of the immune microenvironment (6). Therefore, this study combined different immune subtypes and TLS, follicular regulatory T cells, and fibroblasts, which have a regulatory effect on the tumor immune microenvironment, to evaluate their impact on the efficacy of ICB and identify which immune microenvironment is suitable for ICB.

Immunophenotyping based on immune cell abundance can characterize the immune heterogeneity between tumors, providing a basis for discovering specific immune characteristic populations, especially for identifying potential benefit populations of ICB treatment (17). For example, among the five sarcoma immune classes (SICs) obtained by MCP counter, the SICE subtype not only highly expresses plasma cell genes but also has the highest response rate to pembrolizumab and the longest PFS time (17). In this study, the immunophenotyping based on 64 immune cell abundance inferred from xCell also reflects the heterogeneity of colorectal cancer TIME. Among them, the C3 subtype has potentially ICB-beneficial immune characteristics (high infiltration of CTLs, high expression of PD-L1).

Preclinical investigations into the mechanisms by which various immune cell components participate in the response to anti-PD-1/PD-L1 treatment provide the foundation for our analysis. The TLS and follicular regulatory T cells in tumor tissues have a regulatory effect on the function of TILs in TIME (13). Recent studies have also suggested that TGFβ secreted by fibroblasts is the main mechanism mediating T-cell rejection (11). Fibroblasts are the main type of mesenchymal cells in the tumor microenvironment of colorectal cancer (28). Therefore, in addition to the immunophenotyping, we also included TLS, follicular regulatory T cells, and CAF to comprehensively predict the response to ICB therapy.

From the results of IMvigor210 (**Figure 3** and **Supplementary Figures 2** **,** **3**), the C3 subtype and follicular regulatory T cells may jointly determine the response to ICB treatment. The DHP cohort and GSE94104 consistently revealed that the C3 subtype is featured with the highest expression of TNFRSF9 (**Figures 5**, **7**). TNFRSF9 is the main marker of recently activated regulatory T-cell receptors and follicular regulatory T cells differentiated from such regulatory T cells (12). TIME after nCRT is quite different from untreated tumor tissues (**Figures 4**, **5**, **7**). Among them, the high activation of macrophages and other antigen presentation mechanisms is the main feature in tumor tissue after neoadjuvant therapy. In fact, research has shown that MSI-H colorectal cancer patients with a high abundance of CD68+CD74+ macrophages can benefit significantly from the treatment of nivolumab and pembrolizumab (27). Our analysis shows that the C3 subtype has a large amount of macrophage infiltration, suggesting that the C3 subtype has a good response to ICB treatment.

Although it is generally believed that nCRT of LARC can induce hot tumors, there may be great differences in the composition of the immune microenvironment between hot tumors induced by nCRT and untreated hot tumors (6). More and more preclinical studies have shown that the response mechanism of ICB depends not only on the effector T cells themselves but also on the role of antigen-presenting cells such as macrophages and dendritic cells (27, 29). Therefore, the mechanism of macrophages and their intercellular communication with other immune cells by which ICB is effective after nCRT treatment of LARC needs to be further studied.

There are still many limitations to our study. First of all, due to the lack of expression profiles of patients with LARC treated with immunotherapy, we have only validated the predictive role of immunophenotyping for ICB through the bladder cancer and melanoma databases. Moreover, the targets of anti-PD-L1, anti-PD-1, and anti-CTLA-4 treatment are different; their response mechanisms are not exactly the same (12, 29). Secondly, the immune microenvironment of rectal cancer after nCRT depends not only on the characteristics of the tumor itself but also on the sampling time after nCRT. There are differences in the composition of immune cells sampled at different times after nCRT (6), which raises a problem relating to the window of opportunity for the benefit of specific ICB treatment. We found that the C2 subtype had the shortest RFS in GSE8721 (**Figure 3A**), which was consistent with previous studies on CAF involvement in radiotherapy resistance (30). C3 had the best efficacy of ICB (**Figures 3B, C**). These results preliminarily suggest that immunophenotyping can be used as a factor to predict the efficacy of ICB. However, from the perspective of guiding the individualized treatment of ICB, our analysis did not achieve the purpose of revealing that different immune subtypes need different ICB treatment schemes. In addition, although we used the prediction method based on immune cell infiltration abundance and quality control approach proposed by Petitprez et al. (22), 15.3%–33.3% of the samples in each cohort could not be successfully classified, mainly due to a low Pearson correlation coefficient with the four original subtypes, indicating that these samples may not belong to any of the four subtypes. It does not rule out that the immunophenotyping constructed in early colon cancer is not suitable for local advanced rectal cancer.

In conclusion, we comprehensively analyzed the predictive effect of immune types and the expression profiles of TLS, follicular regulatory T cells, and CAF on the efficacy of ICB treatment. The results suggest that these factors may be coordinated to predict the efficacy of ICB. Our present analysis indicates transcriptomic profiles can provide further biomarkers to stratify rectal cancer patients after nCRT in terms of their response to ICB treatment beyond tumor microsatellite instability status and tumor mutational burden.

## Data availability statement

The date presented in the study are deposited in the GEO repository, accession number GSE213331.

## Ethics statement

The studies involving human participants were reviewed and approved by the ethics committee of Daping Hospital. Written informed consent for participation was not required for this study in accordance with the national legislation and the institutional requirements.

## Author contributions

Conceptualization, HX and DW. Data curation, DJ, YF, and DW. Formal analysis, MJ, BY, and DJ. Funding acquisition, DW. Investigation, LH, MJ, DJ, BY, and ND. Methodology, BY, LH, MJ, and HX. Project administration, DW. Resources, DJ, ND, and YF. Software, BY and LH. Supervision, YF and DW. Validation, LH, ND, and HX. Visualization, LH and HX. Writing—original draft, MJ and HX. Writing—review and editing, DW. All authors contributed to the article and approved the submitted version.

## Funding

The study is supported by the National Key Research and Development Project (2018YFC0114402).

## Conflict of interest

The authors declare that the research was conducted in the absence of any commercial or financial relationships that could be construed as a potential conflict of interest.

## Publisher’s note

All claims expressed in this article are solely those of the authors and do not necessarily represent those of their affiliated organizations, or those of the publisher, the editors and the reviewers. Any product that may be evaluated in this article, or claim that may be made by its manufacturer, is not guaranteed or endorsed by the publisher.
