# Supplementary figures and images for: Identification of four immune subtypes in locally advanced rectal cancer treated with neoadjuvant chemotherapy for predicting the efficacy of subsequent immune checkpoint blockade
Source: Front Immunol. 2022 Sep 27;13:955187. doi: 10.3389/fimmu.2022.955187 (PMC9551659; doi:10.3389/fimmu.2022.955187)

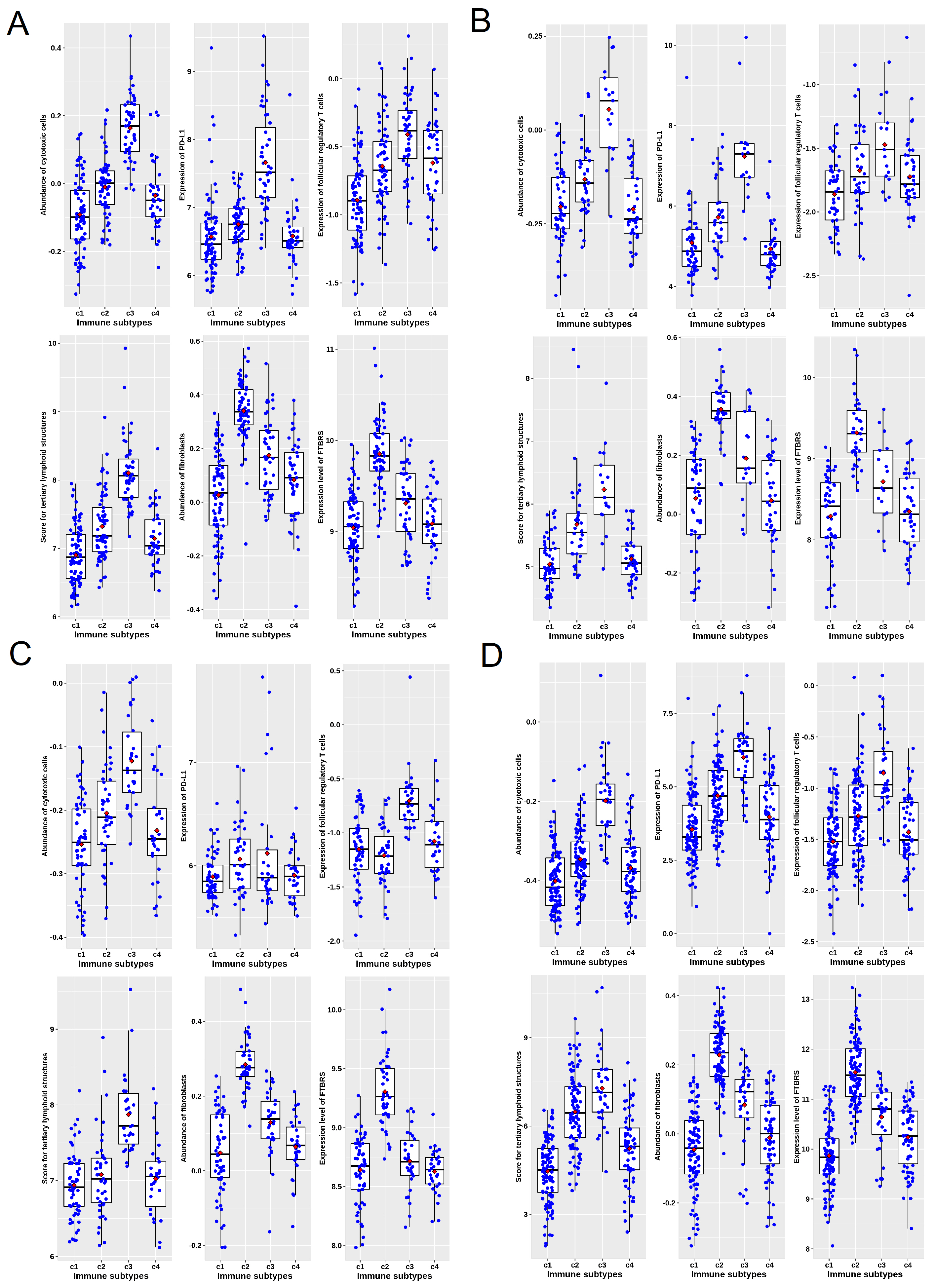

Supplement: Supplementary file 1 [file Image_1.jpeg]

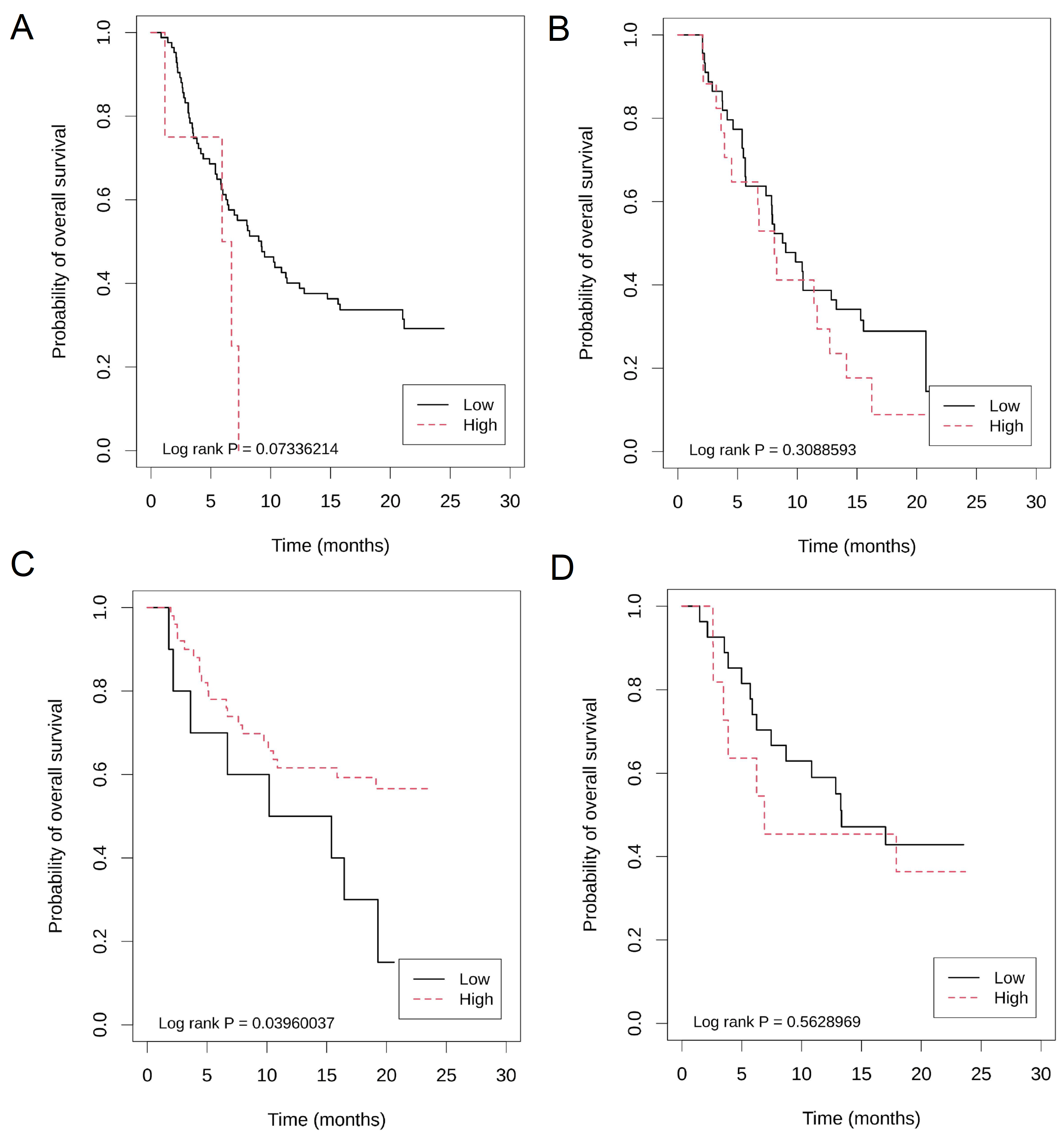

Supplement: Supplementary file 2 [file Image_2.jpeg]

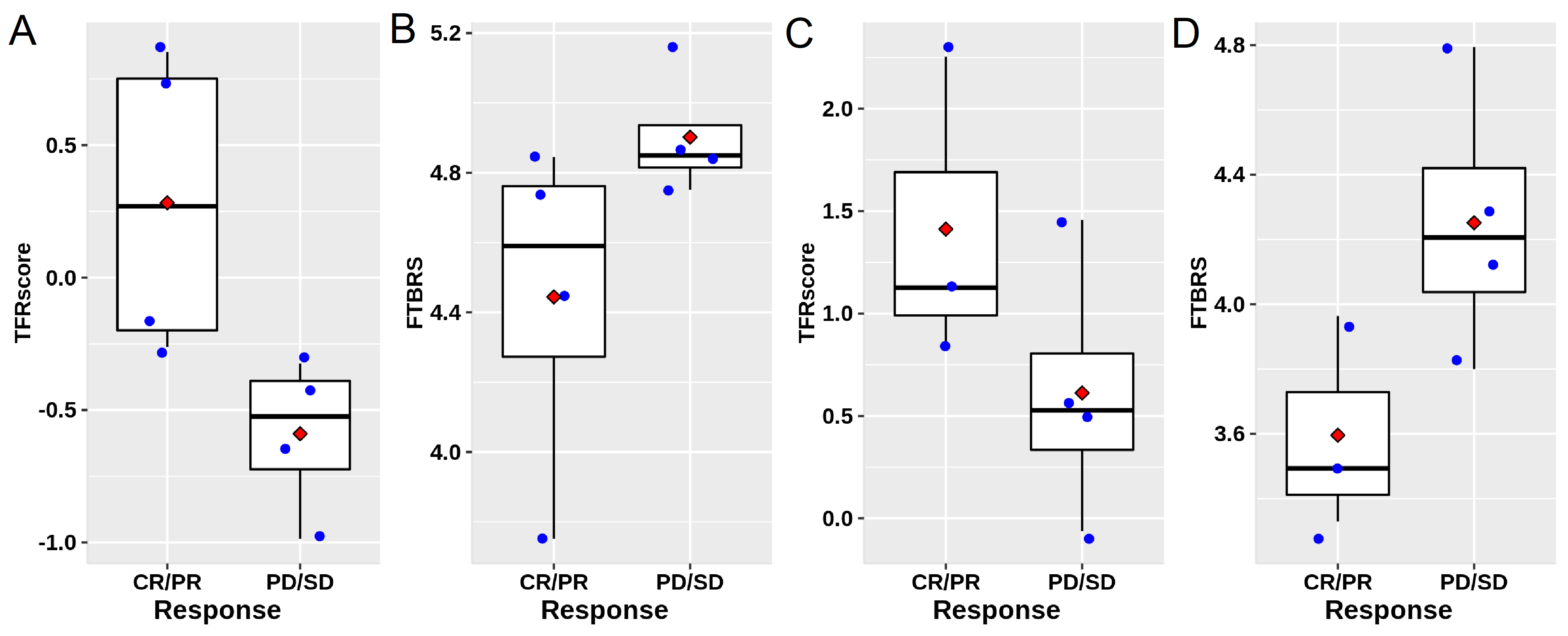

Supplement: Supplementary file 3 [file Image_3.jpeg]
